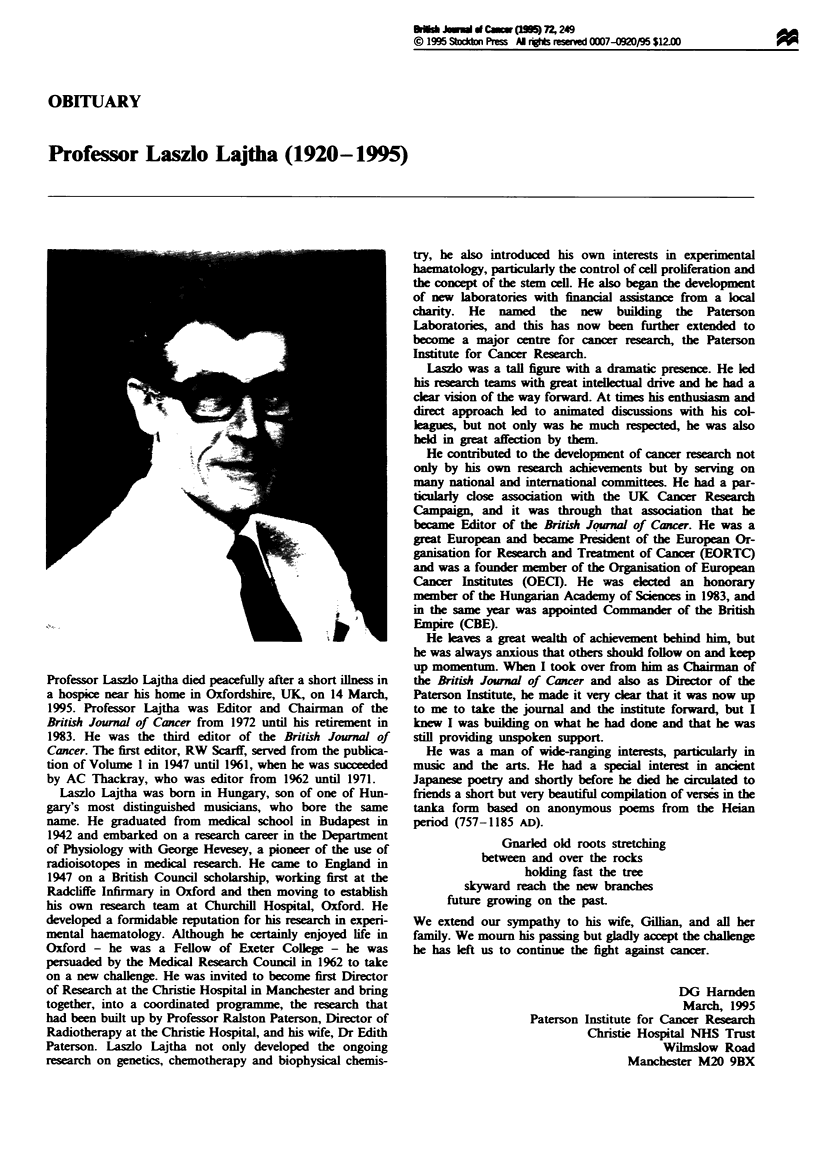# Professor Laszlo Lajtha (1920-1995)

**Published:** 1995-07

**Authors:** DG Harnden

## Abstract

**Images:**


© 1995 Stockton Press All rights reserved 0007-0920/95 $12.00

### OBITUARY

### Professor Laszlo Lajtha (1920–1995)

Professor Laszlo Lajtha died peacefully after a short illness in a hospice near his home in Oxfordshire, UK, on 14 March, 1995. Professor Lajtha was Editor and Chairman of the *British Journal of Cancer* from 1972 until his retirement in 1983. He was the third editor of the *British Journal of Cancer*. The first editor, RW Scarff, served from the publication of Volume 1 in 1947 until 1961, when he was succeeded by AC Thackray, who was editor from 1962 until 1971.

Laszlo Lajtha was born in Hungary, son of one of Hungary's most distinguished musicians, who bore the same name. He graduated from medical school in Budapest in 1942 and embarked on a research career in the Department of Physiology with George Hevesey, a pioneer of the use of radioisotopes in medical research. He came to England in 1947 on a British Council scholarship, working first at the Radcliffe Infirmary in Oxford and then moving to establish his own research team at Churchill Hospital, Oxford. He developed a formidable reputation for his research in experimental haematology. Although he certainly enjoyed life in Oxford – he was a Fellow of Exeter College – he was persuaded by the Medical Research Council in 1962 to take on a new challenge. He was invited to become first Director of Research at the Christie Hospital in Manchester and bring together, into a coordinated programme, the research that had been built up by Professor Ralston Paterson, Director of Radiotherapy at the Christie Hospital, and his wife, Dr Edith Paterson. Laszlo Lajtha not only developed the ongoing research on genetics, chemotherapy and biophysical chemistry, he also introduced his own interests in experimental haematology, particularly the control of cell proliferation and the concept of the stem cell. He also began the development of new laboratories with financial assistance from a local charity. He named the new building the Paterson Laboratories, and this has now been further extended to become a major centre for cancer research, the Paterson Institute for Cancer Research.

Laszlo was a tall figure with a dramatic presence. He led his research teams with great intellectual drive and he had a clear vision of the way forward. At times his enthusiasm and direct approach led to animated discussions with his colleagues, but not only was he much respected, he was also held in great affection by them.

He contributed to the development of cancer research not only by his own research achievements but by serving on many national and international committees. He had a particularly close association with the UK Cancer Research Campaign, and it was through that association that he became Editor of the *British Journal of Cancer*. He was a great European and became President of the European Organisation for Research and Treatment of Cancer (EORTC) and was a founder member of the Organisation of European Cancer Institutes (OECI). He was elected an honorary member of the Hungarian Academy of Sciences in 1983, and in the same year was appointed Commander of the British Empire (CBE).

He leaves a great wealth of achievement behind him, but he was always anxious that others should follow on and keep up momentum. When I took over from him as Chairman of the *British Journal of Cancer* and also as Director of the Paterson Institute, he made it very clear that it was now up to me to take the journal and the institute forward, but I knew I was building on what he had done and that he was still providing unspoken support.

He was a man of wide-ranging interests, particularly in music and the arts. He had a special interest in ancient Japanese poetry and shortly before he died he circulated to friends a short but very beautiful compilation of verses in the tanka form based on anonymous poems from the Heian period (757–1185 AD).

Gnarled old roots stretching
between and over the rocks
holding fast the tree
skyward reach the new branches
future growing on the past.

We extend our sympathy to his wife, Gillian, and all her family. We mourn his passing but gladly accept the challenge he has left us to continue the fight against cancer.

DG Harnden
March, 1995
Paterson Institute for Cancer Research
Christie Hospital NHS Trust
Wilmslow Road
Manchester M20 9BX